# Combination of miRNA499 and miRNA133 Exerts a Synergic Effect on Cardiac Differentiation

**DOI:** 10.1002/stem.1928

**Published:** 2015-03-24

**Authors:** Federica Pisano, Claudia Altomare, Elisabetta Cervio, Lucio Barile, Marcella Rocchetti, Maria Chiara Ciuffreda, Giuseppe Malpasso, Francesco Copes, Manuela Mura, Patrizia Danieli, Gianluca Viarengo, Antonio Zaza, Massimiliano Gnecchi

**Affiliations:** a Department of Cardiothoracic and Vascular Sciences—Coronary Care Unit and Laboratory of Clinical and Experimental Cardiology, Fondazione IRCCS Policlinico San MatteoPavia, Italy; b Laboratory of Experimental Cardiology for Cell and Molecular Therapy, Fondazione IRCCS Policlinico San MatteoPavia, Italy; e Division of Clinical Immunology, Immunohematology and Transfusion Service, Fondazione IRCCS Policlinico San MatteoPavia, Italy; c Department of Biotechnology and Biosciences, University of Milano-BicoccaMilan, Italy; d Department of Molecular Medicine, Unit of Cardiology, University of PaviaPavia, Italy; f Department of Medicine, University of Cape TownCape Town, South Africa

**Keywords:** Cardiac differentiation, P19 cells, Stem cells, MicroRNA, Electrophysiology

## Abstract

Several studies have demonstrated that miRNA are involved in cardiac development, stem cell maintenance, and differentiation. In particular, it has been shown that miRNA133, miRNA1, and miRNA499 are involved in progenitor cell differentiation into cardiomyocytes. However, it is unknown whether different miRNA may act synergistically to improve cardiac differentiation. We used mouse P19 cells as a cardiogenic differentiation model. miRNA499, miRNA1, or miRNA133 were transiently over-expressed in P19 cells individually or in different combinations. The over-expression of miRNA499 alone increased the number of beating cells and the association of miRNA499 with miRNA133 exerted a synergistic effect, further increasing the number of beating cells. Real-time polymerase chain reaction showed that the combination of miRNA499 + 133 enhanced the expression of cardiac genes compared with controls. Western blot and immunocytochemistry for connexin43 and cardiac troponin T confirmed these findings. Importantly, caffeine responsiveness, a clear functional parameter of cardiac differentiation, was increased by miRNA499 in association with miRNA133 and was directly correlated with the activation of the cardiac troponin I isoform promoter. Cyclic contractions were reversibly abolished by extracellular calcium depletion, nifedipine, ryanodine, and IP3R blockade. Finally, we demonstrated that the use of miRNA499 + 133 induced cardiac differentiation even in the absence of dimethyl sulfoxide. Our results show that the areas spontaneously contracting possess electrophysiological and pharmacological characteristics compatible with true cardiac excitation-contraction coupling. The translational relevance of our findings was reinforced by the demonstration that the over-expression of miRNA499 and miRNA133 was also able to induce the differentiation of human mesenchymal stromal cells toward the cardiac lineage. Stem Cells *2015;33:1187–1199*

## Introduction

Cardiovascular diseases are the major cause of morbidity and mortality worldwide, with heart failure representing one of the most severe clinical manifestation [1,2]. Congestive heart failure involves loss of cardiac tissue, deposition of fibrotic tissue, and ventricular remodeling, which result in decreased cardiac output and insufficient blood supply to all organs. It is known that ischemic heart disease, in particular myocardial infarction (MI), is the first cause of heart failure. Based on several lines of evidence that after ischemic damage cardiomyocytes (CMC) are replenished, albeit at extremely low levels, stem cell therapy to regenerate cardiac tissue has been proposed as a possible strategy to treat MI [3–6]. Although encouraging results have been obtained in experimental models, the efficiency of cardiac regeneration is very poor, with paracrine effects playing a major role in mediating the positive effects observed [7–11]. Notably, the primary barrier to progress in this area of research is tied to the fact that the capacity of adult stem cells to differentiate into mature CMC remains an enigma [12]. Therefore, in order to achieve true myocardial regeneration it will be necessary to discover new strategies to improve the differentiation process.

The well-characterized multipotent embryonic cell line P19 was chosen as a platform for this study based on its established capacity to differentiate into CMC. It has been shown that the rhythmic beating of P19-derived CMC is paralleled by the development of a fully operational contractile apparatus [13]. Moreover, electrophysiological studies performed on CMC derived from P19 cells demonstrated the presence of Na^+^, Ca^2+^, and K^+^ channels that play a pivotal role during the contraction process [14]. Finally, chronotropic effects were found for adrenaline, phenylephrine, and forskolin indicating the involvement of regulatory actions by α- and β-adrenoceptors and adenylyl cyclase-dependent regulation in beating frequency [15].

Several studies have recently demonstrated that miRNA are involved in cardiac development, stem cell maintenance, and differentiation via translational repression [16]. In particular, it has been clearly shown that miRNA133 and miRNA1 promote myoblast proliferation and differentiation, respectively, and that miRNA499 enhances the differentiation of cardiac progenitor cells into CMC [17–20]. However, it is currently unknown whether these miRNA may act synergistically. Accordingly, we tested this hypothesis by over-expressing different combinations of miRNA1, 133, and 499 in P19 cells, which are considered an ideal model to study cardiac differentiation in vitro. Furthermore, to reinforce the translational relevance of our finding, we tested the same approach on human amniotic mesenchymal stromal cells (AMSC).

## Materials and Methods

### P19 Cell Culture

P19 cells were purchased from IZSLER (Istituto Zooprofilattico Sperimentale, Lombardy and Emilia Romagna “Bruno Ubertini”, Brescia, Italy, http://www.izsler.it/) and were grown in alpha-minimum essential medium (α-MEM, Celbio Euroclone Group, Milan, Italy, http://www.euroclonegroup.it/) supplemented with 10% fetal bovine serum (Sigma-Aldrich, St. Louis, MO https://www.sigmaaldrich.com/), 2 mM l-glutamine (Gibco, Invitrogen, Life Technologies, Carlsbad, CA, http://www.lifetechnologies.com/it/en/home/brands/invitrogen.html), 50 U/ml penicillin, and 50 μg/ml streptomycin (Gibco, Invitrogen, Life Technologies) in a 5% CO_2_ atmosphere at 37 °C. Confluent cells were split (1:3–1:5 ratio) by trypsinization. Cells were negative when tested for mycoplasma contamination with the e-Myco polymerase chain reaction (PCR) detection kit (iNtRON Biotechnology, Sangdaewon-dong, Korea, http://eng.intronbio.com/).

### P19 Transduction with Cardiac Troponin I Isoform

To better monitor cardiac differentiation over time, before proceeding with the cardiac differentiation protocol, P19 cells were transduced with a lentivirus in which green fluorescent protein (GFP) expression is controlled by a cardiac-specific promoter, namely cardiac troponin I isoform (cTnI). The plasmid carrying the specific cardiac promoter was a kind gift from Prof. Gianluigi Condorelli [21] (Supporting Information Fig. S1). The virus was used at a multiplicity of infection (MOI) of 3 TU/ml in the presence of 8 μg/ml polybrene (Sigma-Aldrich). Transduction was achieved without impairing adhesion, growth, or proliferation of P19 cells. To evaluate the transduction efficiency, PCR and Western blot (WB) were performed on transduced cells. In detail, RNA and proteins were isolated from the cells. Total RNA was extracted with Trizol Reagent (Invitrogen, Life Technologies) according to the manufacturer's instructions. After quantification, 500 ng of RNA was reverse transcribed with SuperScript II Reverse Transcriptase (Invitrogen, Life Technologies) and the cDNA was used as a template for the PCR assay. Specific primers for the GFP gene (Supporting Information Table S1) were used to detect the presence of the transgenic tag sequence. Proteins were extracted with RIPA buffer and analyzed by WB (Supporting Information Fig. S2).

### Embryoid Body Formation and Differentiation

To induce cardiac differentiation, 4 ×10^5^ P19 cells were cultured in suspension in 100 mm bacteriological Petri dishes in the presence of control (CTRL) medium or medium supplemented with 0.5% dimethyl sulfoxide (DMSO) (differentiation medium). Under these conditions, P19 cells form embryoid bodies (EB). After 4 days, the EB were transferred to plastic culture dishes in the presence of differentiation medium, and transfected with precursor molecules (pre-miRNA) for miRNA499 (PM11352, 10 nM), miRNA1 (PM10617, 10 nM), and miRNA133 (PM10413, 5 nM) in different combinations or with scrambled miRNA used as a negative CTRL (AM17110, 5 nM) (Supporting Information Table S1). All pre-miRNA molecules were purchased from Ambion (Life Technologies, Carlsbad, CA, http://www.lifetechnologies.com/it/en/home/brands/ambion.html). EB were transfected with siPORT NeoFX Transfection Agent following the manufacturer's protocol (Ambion, Life Technologies). In additional experiments, to test whether miRNA alone can trigger cardiac differentiation, P19 were maintained in Petri dishes in CTRL medium without 0.5% DMSO during the EB formation, and later transferred to adherent culture plates for transfection with pre-miRNA.

### Transfection of Embryoid Body with miRNA Precursors

EB were collected using a pipette and transferred to 50 ml falcon tubes. After EB settled, the supernatants were aspirated and fresh complete medium was added. For each 100 mm Petri dish, EB were resuspended in 14.4 ml of medium. The transfection agent siPORT NeoFX was diluted in Opti-MEM I medium (Gibco, Invitrogen, Life Technologies) and left for 10 minutes at room temperature. miRNA precursors were diluted in Opti-MEM I medium at the following concentration: miRNA1 and miRNA499 precursors 10 nM, miRNA133 precursor and scrambled miRNA 5 nM. After incubation, the mix with siPORT NeoFX was added to the mix containing the diluted miRNA precursors and incubated for an additional 10 minutes at room temperature. Then, the 14.4 ml of medium containing the EB was collected and aliquots of 2.4 ml seeded in each well of a six multiwell plate. The plates were gently rocked back and forth and incubated over-night at 37 °C in 5% CO_2_. The next day, the medium was changed with fresh complete medium and cells attached to multiwells were maintained in culture for the differentiation protocol.

### Quantitative Analysis of Beating Clusters

The number of beating clusters was counted 14 days after the induction of differentiation. Briefly, EB exposed to different treatments were cultured in six-well plates and medium was replaced every 3 days. We drew a grid on the bottom of the plate and the beating clusters in the central four areas were blindly counted.

### Evaluation of cTnI Expression

To quantify the expression of cTnI, we observed the EB with a fluorescent microscope (Zeiss Axio Observer Z1, Zeiss, Oberkochen, Germany, http://www.zeiss.com/corporate/en_de/global/home.html) at different time points. At day 14, cells were trypsinized, washed three times in phosphate buffered saline (PBS), and then counted by fluorescent-activated cell sorting (FACS) with a FACSCalibur flow cytometer (BD Biosciences, San Jose, CA, http://www.bdbiosciences.com/) after 14 days differentiation.

### MEA Measurements

Beating foci of P19-derived EB were dissected and plated in culture medium on 0.1% gelatin-Matrigel coated multi-electrode array (MEA) dishes for 2 days. Spontaneous field potentials were recorded at physiological temperature using MEA256 (Multi Channel Systems, Reutlingen, Germany, http://www.multichannelsystems.com/) at a sampling rate of 20 kHz and low-pass filtered at 5 kHz. Tetrodotoxin (30 μM TTX, Tocris, Bristol, U.K. http://www.tocris.com/), barium chloride (1 mM Ba^2+^, Sigma-Aldrich), and nifedipine (5 μM, Sigma-Aldrich) were added to culture medium during measurements.

### Twitch Measurements

Spontaneous mechanical activity (unstimulated twitch) of P19-derived EB was optically recorded by a Video Edge Detection System. EB were plated on coverslips and left to expand as cell layers. Detection cursors were placed on the edges of contracting portions of the preparations, which were superfused with Tyrode solution containing (in mM): 154 NaCl, 4 KCl, 2 CaCl_2_, 1 MgCl_2_, 5 HEPES NaOH, 5.5 glucose, pH 7.35 (*T* = 35 °C ± 0.5 °C). Test conditions included Ca^2+^ removal (0 Ca^2+^ + 1 mM EGTA), nifedipine (5 μM), ryanodine (30 μM), or 2-aminoethoxydiphenyl borate 10 μM (2-APB). All reagents were purchased from Sigma-Aldrich.

### [Ca^2+^]_I_ Measurements

Cells were incubated with the Ca^2+^-sensitive dye, Fluo4-AM 10 μM (Molecular Probes, Invitrogen, Life Technologies, Carlsbad, CA, http://www.lifetechnologies.com/it/en/home/brands/molecular-probes.html) for approximately 45 minutes at room temperature and washed with Tyrode solution for approximately 15 minutes. Changes in intracellular Ca^2+^ were recorded with a laser-scanning confocal microscope (Leica TCS SP2, Leica, Solms, Germany, http://www.leica.com/); Fluo4-AM was excited with an argon laser at *λ* = 488 nm and the emission fluorescence (*F*) was detected at *λ* > 512 nm (Leica Confocal Software). Fluorescence two-dimensional images (*xy*, 512 × 512 pixels, optical thickness = 1 μm) were sampled every 0.8 seconds in low magnification fields containing cell populations. Image processing was performed off-line by the ImageJ software. Pixels corresponding to cell-covered areas of the field (“cell pixels”) were identified with a built-in algorithm based on the distribution of pixel values in a “sum-image” constructed by summing all frames of the field obtained throughout the experiment. The ratio between *F* over time and its mean value under basal conditions (*F*/*F*0) was recorded for each frame and used to quantify Ca^2+^ changes over time. Cells were challenged with caffeine 10 mM to release intracellular Ca^2+^ stores; the measurement was repeated in the presence of the SERCA inhibitor cyclopiazonic acid (CPA 50 μM) or ryanodine. Responsiveness to caffeine was evaluated in each field by counting the number of pixels in which *F*/*F*0 exceeded 2 SD basal *F*/*F*0 (“active pixels”) and dividing it by the number of cell pixels.

### Statistical Analysis

Data are presented as mean ± SD of at least three independent experiments. The statistical significance of differences in mean values among comparison groups was determined by one-way analysis of variance (ANOVA) after assumptions of normal distribution and of homogeneity of variances were verified or, in case of non normal distribution, by Kruskal-Wallis. Differences were considered statistically significant when the *p* value was inferior to.05. After a significant result from ANOVA was obtained, Bonferroni's correction for multiple testing was applied, producing the significance level reported.

## Results

### Pre-miRNA Stimulates P19 Cells to Differentiate into CMC

**Figure 1:**
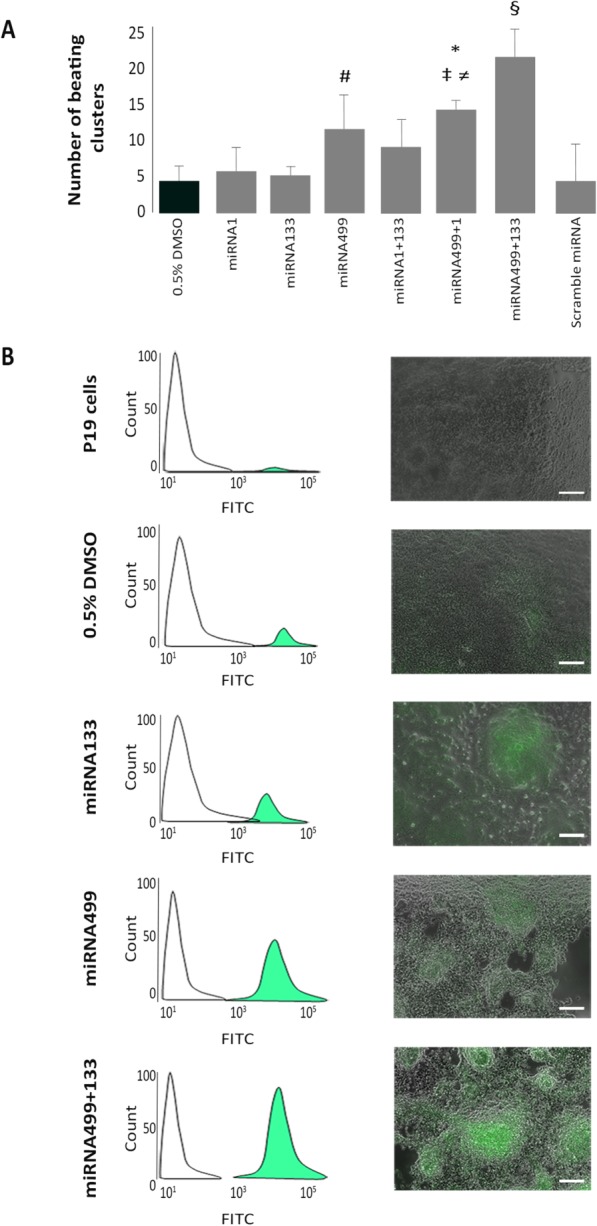
Quantification of beating clusters. (A): Number of contracting embryoid bodies (EB) under different conditions. (#, *p* < .001 vs. DMSO and miRNA133; *, *p* < .001 vs. DMSO, miRNA1 and miRNA133; ‡, *p* < .01 vs. scramble miRNA; ≠, *p* < .05 vs. miRNA1 + 133, §, *p* < .001 vs. all conditions). (B): Fluorescence-activated cell sorting analysis of green fluorescent protein positive EB derived from P19 cells CTRL (0.3%) and treated with 0.5% DMSO (2.3%), miRNA133 (7.2%), miRNA499 (43.8%), or miRNA499+miRNA133 (79.2%). Scale bar = 100 μm.

In order to verify whether the coexpression of different miRNA plays a procardiogenic effect, the number of beating EB was counted and in parallel the expression of cTnI was quantified during the first 14 days of culture. At day 14, the over-expression of miRNA1 or miRNA133 alone or their combination did not increase the number of beating clusters compared with DMSO treatment (Fig. 1A). On the contrary, pretreatment with miRNA499 alone significantly increased the number of beating clusters compared with DMSO (+2.1-fold; *p* < .001) (Fig. 1A). By simultaneously over-expressing miRNA499 and miRNA1, the number of beating EB significantly increased compared with: DMSO (+2.8-fold; *p* < .001), miRNA1 (+2.5-fold; *p* < .001), and miRNA133 (+2.7-fold; *p* < .001), but not compared with miRNA499 alone (*p* = NS). Pretreatment of P19 cells with both miRNA499 and miRNA133 markedly increased the number of beating clusters compared with all the other conditions tested. In particular, the increase was 4.3-fold versus DMSO (*p* < .001), 4.1-fold versus miRNA133 alone (*p* < .001), and 2-fold versus miRNA499 alone (*p* < .001), suggesting a relevant and synergistic effect of these two miRNAs in driving cardiac differentiation (Fig. 1A).

The synergistic effect exerted by the combination of miRNA133 and miRNA499 was confirmed by activation of the cTnI cardiac-specific promoter (Fig. 1B). Undifferentiated P19, as expected, did not express GFP, while treatment with DMSO turned a certain number of clusters green (Fig. 1B). The treatment of EB with both pre-miRNA499 and pre-miRNA133 resulted in the strongest activation of the cTnI promoter (Fig. 1B). Furthermore, daily observation of our clusters showed that treatment with pre-miRNA499 plus pre-miRNA133 anticipated the activation of the cTnI promoter compared with all other conditions (data not shown). The results acquired by fluorescence microscopy were confirmed by FACS analysis. Treatment with both miRNA499 and miRNA133 activated 79.2% of the cells compared with 2.3% of GFP^+^ cells after DMSO treatment, 7.2% with miRNA133 alone, and 43.8% with miRNA499 alone (Fig. 1B). These data strongly suggest a synergistic effect of miRNA499 and miRNA133.

### The Combination of miRNA499 and miRNA133 Increases the Expression of Cardiac-Specific Genes

**Figure 2:**
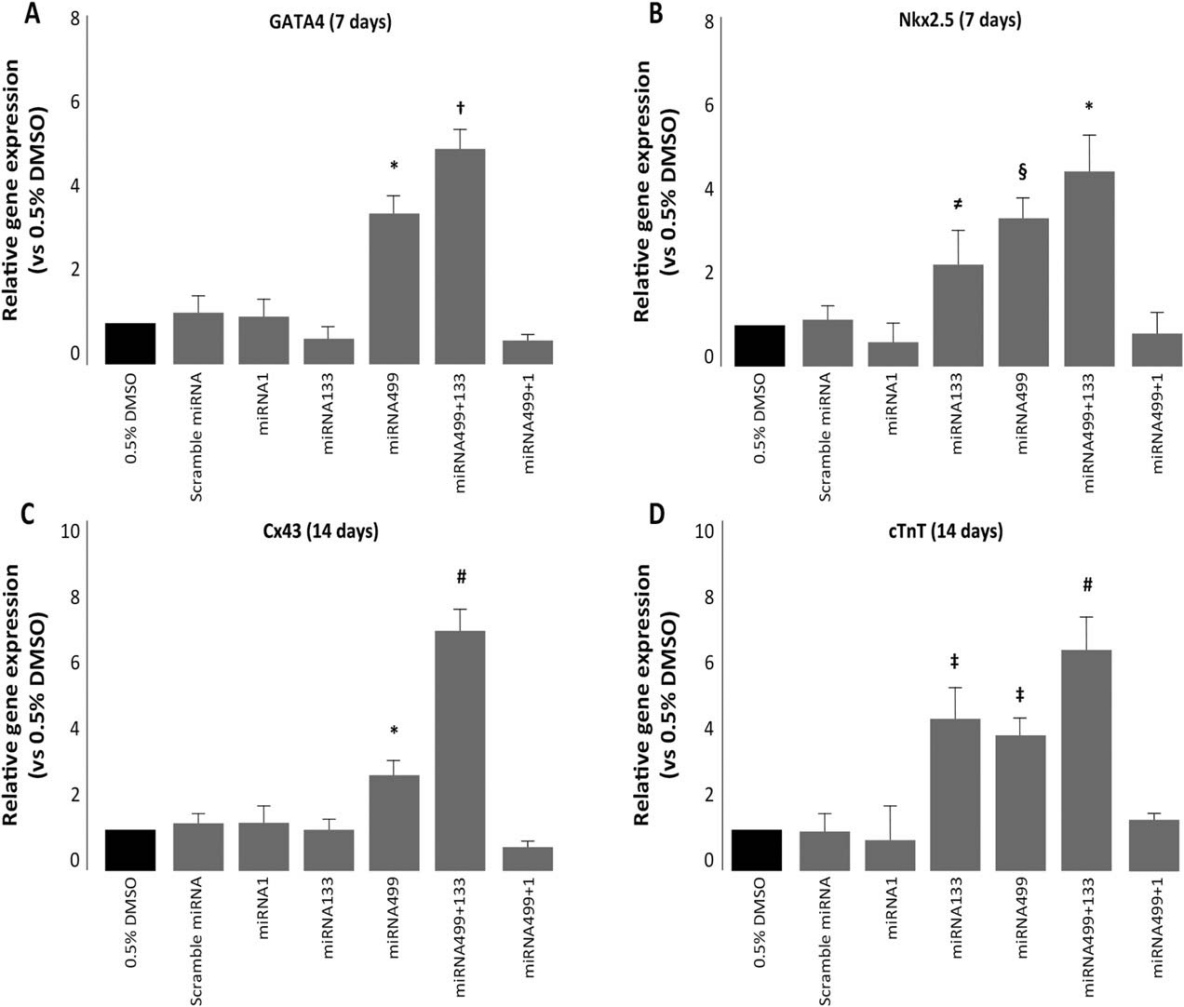
Expression of cardiac-specific genes. Real-time polymerase chain reaction of the “early” cardiac genes GATA4 (A) and Nkx2.5 (B) analyzed at 7 days and “late” cardiac genes Cx43 (C) and cTnT (D) analyzed at 14 days (*, *p* < .001 vs. DMSO, scramble miRNA, miRNA1, miRNA133, and miRNA499 + 1; †, *p* < .001 vs. DMSO, scramble miRNA, miRNA1, miRNA133, and miRNA499 + 1, and *p* < .01 vs. miRNA499; ≠, *p* < .001 vs. miRNA1 and *p* < .01 vs. DMSO, scramble miRNA, miRNA499 + 1; §, *p* < .001 vs. DMSO, scramble miRNA, miRNA1, and miRNA499 + 1; #, *p* < .001 vs. all conditions; ‡, *p* < .001 vs. DMSO, scramble miRNA, miRNA1, and miRNA499 + 1).

The expression of cardiac-specific genes was quantified by real-time PCR after 7 or 14 days of culture. In particular, we quantified “early” cardiac genes such as GATA4 and Nkx2.5 at 7 days and “late” cardiac genes at 14 days. The expression of both GATA4 and Nkx2.5 was significantly increased by miRNA499 alone (Fig. 2A, 2B). miRNA133 increased the expression of Nkx2.5 (+1.3-fold vs. 0.5% DMSO and scramble, *p* < .01; +1.5-fold vs. miRNA499 + 1, *p* < .01) (Fig. 2B), but had no effect on GATA4 (Fig. 2A), while miRNA1 triggered the expression of neither GATA4 nor Nkx2.5 (Fig. 2A, 2B). When miRNA499 and miRNA133 were coexpressed, we documented a significant increase in both GATA4 and Nkx2.5 expression compared with all other conditions tested (Fig. 2A, 2B). In particular, compared with DMSO, GATA4 was 4.7-fold higher after treatment with the combination of pre-miRNA499 and pre-miRNA133 (*p* < .001), and Nkx2.5 was 4.2-fold higher (*p* < .001).

After 14 days, quantification of late cardiac-specific genes confirmed the synergistic effect exerted by miRNA499 and miRNA133 (Fig. 2C, 2D). Indeed, only in P19 cells treated with this combination we observed a significant increase in the expression of both Cx43 and cTnT compared with all other conditions tested (Fig. 2C, 2D). In particular, compared with DMSO, Cx43 was increased 7-fold (*p* < .001) and cTnT 6.2-fold (*p* < .001). Also miRNA499 alone increased the expression of both Cx43 (+3.1-fold vs. DMSO; *p* < .001) and cTnT (+3.4-fold vs. DMSO; *p* < .001), but to a significantly lower extent compared with miRNA499 and miRNA133 in combination (Fig. 2C, 2D). The over-expression of miRNA1 alone or in association with miRNA499 failed to increase the expression level of the cardiac-specific differentiation markers considered.

To better characterize the nature of the cardiac-like cells, we quantified the expression of the atrial marker Mlc.2A and the ventricular marker IRX4. The over-expression of miRNA499 resulted in the upregulation of both genes compared with DMSO (Supporting Information Fig. S3A, S3B). The coexpression of miRNA499 and miRNA133 further increased the expression of the atrial marker Mlc.2A (Supporting Information Fig. S3A). Treatment of P19 cells with miRNA499 or miRNA499 + 133 increased also the expression of the ventricular marker IRX4 (Supporting Information Fig. S3B).

### Cardiac-Specific Proteins Are Highly Expressed in P19 Cells Treated with miRNA499 and miRNA133

**Figure 3:**
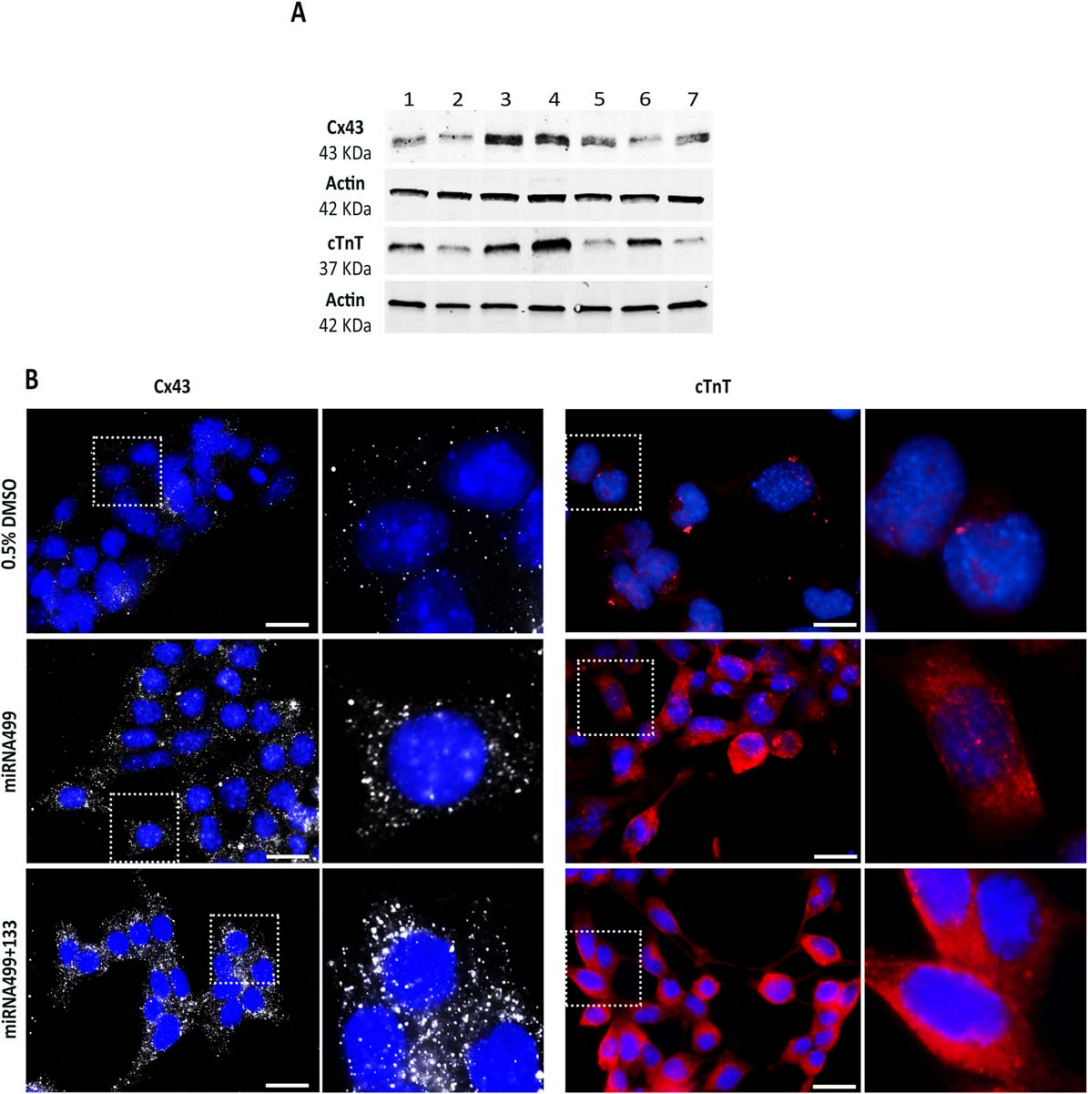
Protein analysis by WB and ICC. (A): Western blot analysis for Cx43 and cTnT of embryoid bodies treated with miRNA133 (1), miRNA1 (2), miRNA499 (3), miRNA499 + 133 (4), miRNA499 + 1 (5), 0.5% DMSO alone (6), or scramble miRNA (7). (B): ICC for Cx43 (white) and cTnT (red) after treatment with 0.5% DMSO, miRNA499, or miRNA499 plus miRNA133. High magnification images of the areas delimited by dotted square are also reported. Scale bar = 25 μm.

To confirm the procardiogenic effect exerted by miRNA over-expression at the protein level, we verified the expression of Cx43 and cTnT by WB and immunocytochemistry (ICC) (Fig. 3A, 3B). At the 14 days time point, WB showed that miRNA1 alone had no effect on both Cx43 and cTnT, miRNA133 increased only the expression of cTnT, while miRNA499 was able to markedly increase the expression of both Cx43 and cTnT (Fig. 3A). The combination of miRNA499 and 133 greatly enhanced the expression of both Cx43 and cTnT (Fig. 3A). On the contrary, the coexpression of miRNA1 with miRNA499 did not increase the expression of the two cardiac-specific proteins compared with miRNA499 alone (Fig. 3A).

ICC experiments confirmed that Cx43 and cTnT were convincingly turned on upon over-expression of miRNA449 alone and even more so in combination with miRNA133 (Fig. 3B). In particular, coexpression of Cx43 and cTnT was always present in those cells forming beating clusters, confirming that both contractile and channels proteins are present in the EB treated with the combination of miRNA499 and 133.

### Caffeine- or Nicotine-Triggered Ca^2+^ Response

**Figure 4:**
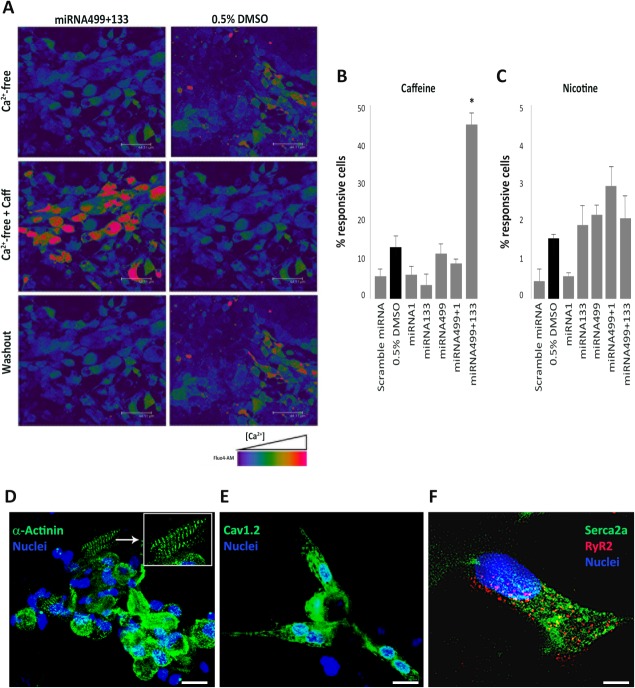
Detection of caffeine-induced Ca^2+^ release and immunocytochemistry. (A): Images captured during recording of FLUO-4AM loaded EB treated with DMSO alone or DMSO plus the combination of miRNA499 and miRNA133 (Supporting Information Video S1). EB were analyzed during superfusion of caffeine or in washout conditions (Ca^2+^-free as control extracellular solution). Changes in intracellular calcium concentration were detected as variation in fluorescence reported by the false-color scale shown on the bottom (red highest). (B, C): Frequency of caffeine (B) and nicotine (C) response (expressed as % of responsive cells) in EB treated with 0.5% DMSO alone or after treatment with different pre-miRNA, alone or in combination (*, *p* < .05 vs. all conditions). (D): ICC of α-sarcomeric actinin (green). (E): ICC of Cav1.2 channels (green). (F): ICC of SERCA2a (green) and RyR2 channels (red). Nuclei were stained with Hoechst33258. Scale bar = 25 μm.

This set of experiments aimed to assess the proportion of cells endowed with a functional RyR-operated (caffeine-releasable) intracellular Ca^2+^ store, a central element of excitation-contraction coupling of mature muscle cells. To rule out Ca^2+^ influx from the extracellular space, caffeine was administered during superfusion with Ca^2+^-free solutions (0 Ca^2+^ plus 1 mM EGTA). EB were seeded on glass coverslips and allowed to expand as cell layers. Beating layers were loaded with the Ca^2+^ sensitive dye, Fluo-4, and the proportion of caffeine-responsive cells for the various treatment groups was quantified as described in Materials and Methods; the effect of 0.5% DMSO, contained in all treatment solutions, was evaluated separately as it represents the CTRL condition (Fig. 4A, Supporting Information Video S1). Coexpression of miRNA499 and miRNA133 induced a 3.5-fold increase in the number of responsive cells with respect to cells exposed to DMSO (*p* < .05), thus indicating enhanced expression of a muscle-type intracellular Ca^2+^ store. Caffeine responsiveness was not significantly increased by any other treatment, thus supporting the specificity of miRNA499 + 133 effect (Fig. 4B).

To verify their dependency on the presence of a functional sarcolplasmic reticulum (SR), caffeine-induced responses were tested after superfusion with the SERCA inhibitor CPA (50 μM) or incubation with ryanodine (30 μM). No caffeine-triggered responses were observed after CPA or ryanodine incubation (Supporting Information Fig. S4).

To exclude differentiation toward skeletal muscle lineage, intracellular Ca^2+^ was measured during challenge with nicotine pulses (100 μM), an agonist of cholinergic receptor-operated channels (nAChR). Although not directly involved in Ca^2+^ transport, nAChR mediates membrane depolarization and the resulting voltage-dependent Ca^2+^ transients [22]. Nicotine failed to induce detectable Ca^2+^ transients in all treatment groups; in particular, we did not observe a significant increase in responsive cells after treatment with miRNA499 and miRNA133 compared to DMSO standard condition (Fig. 4C).

### Expression of Cardiac Excitation/Contraction-Coupling Proteins

Expression of cardiac cytoskeletal protein (α-sarcomeric actinin) and of other important proteins involved in cardiac excitation/contraction (EC)-coupling (Cav1.2, SERCA2a, and RyR2) was analyzed by ICC on EB coexpressing miRNA499 and miRNA133, selected from the same batch of EB showing caffeine-responsiveness. The cells stained positive for α-sarcomeric actinin, which was often organized in a sarcomeric fashion (Fig. 4C). Sarcolemmal Ca^2+^ channels (Cav1.2) (Fig. 4D), the SR Ca^2+^ ATP-ase (cardiac isoform SERCA2a), and SR Ca^2+^ release channel (cardiac isoform RyR2) were also expressed and localized in the same cells of the field (Fig. 4E).

### Mechanisms of Spontaneous Beating in Embryoid Bodies

**Figure 5:**
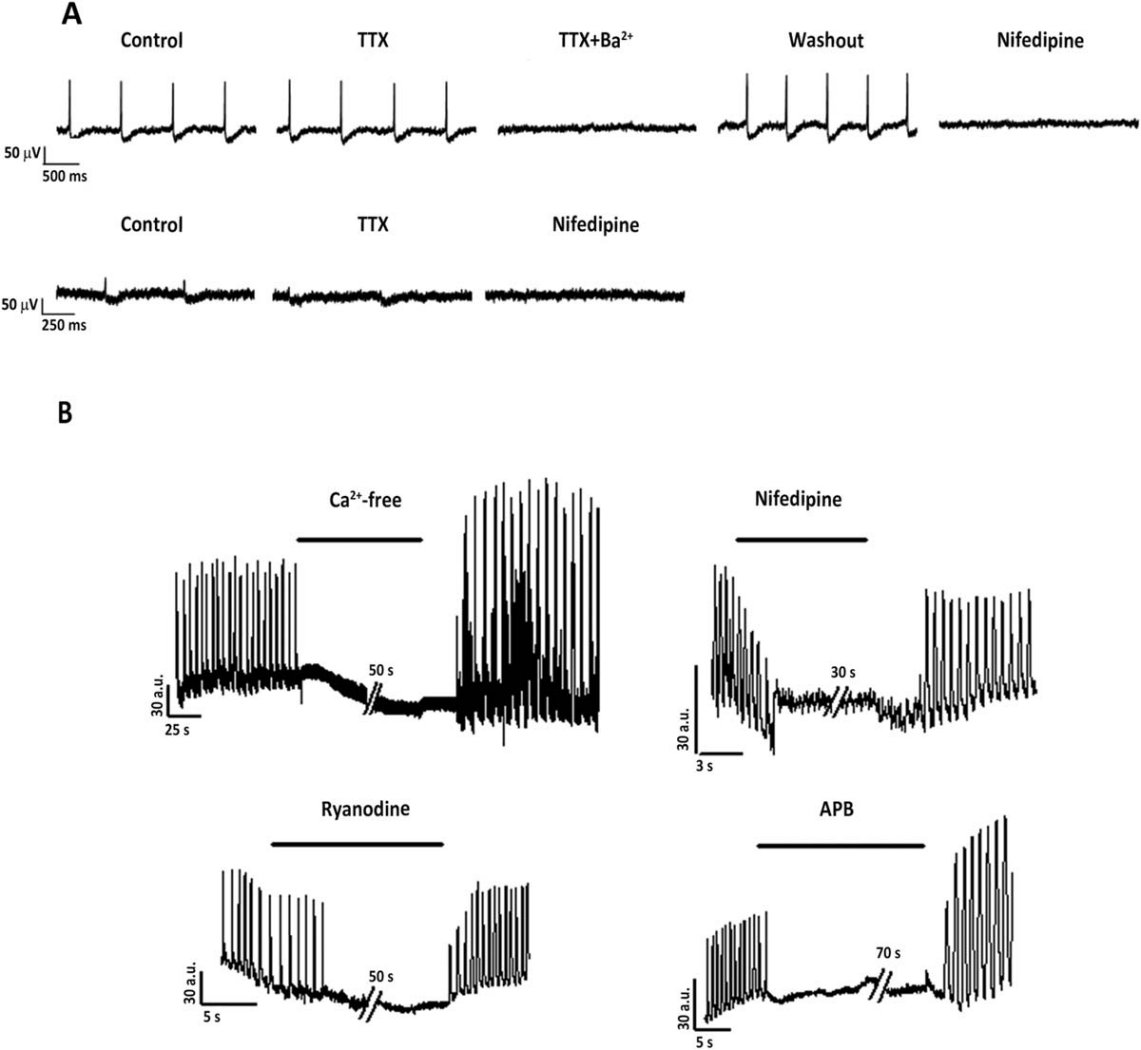
MEA and twitch recordings of embryoid bodies treated with pre-miRNA499 together with pre-miRNA133. (A): Top: example of single MEA electrode field potential generated by a spontaneously beating cluster under control conditions, in the presence of TTX (30 μM, alone or together with Ba^2+^ 1 mM) or nifedipine (5 μM). Bottom: example of a fast component recorded in beating clusters under control conditions or in the presence of TTX (30 μM) or nifedipine (5 μM). Steady-state effects of drugs are shown. (B): Spontaneous contractile activity was analyzed during exposure to Ca^2+^-free solution for 50 seconds, nifedipine for 30 seconds, ryanodine for 50 seconds, and 2-APB for 70 seconds. Abbreviation: TTX, tetrodotoxin; APB, 2-aminoethoxydiphenyl borate.

This set of experiments tested the involvement of membrane ion channels and the SR machinery in the beating EB. Field potentials, generated by spontaneously beating foci, were measured by MEA before and after challenge with specific ion channel blockers. To ensure stability of activity, preparations were monitored for at least 5 minutes in each condition before measurements. Figure 5A (upper panel) shows the electrical activity of a miRNA-treated EB in control conditions and during blockade of Na^+^ (TTX), potassium (Ba^2+^), and L-type Ca^2+^ (nifedipine) channels. While Ba^2+^ and nifedipine abolished spontaneous electrical activity, TTX did not affect it. The same response pattern was observed in six separate control preparations (0.5% DMSO; Supporting Information Fig. S5). In two additional miRNA-treated preparations, TTX abolished the fast electrogram component although it failed to stop firing (Fig. 5a, bottom panel).

To investigate the mechanism of spontaneous mechanical activity of cells showing responsiveness to caffeine, spontaneous contractions were recorded in cell layers. Removal of extracellular Ca^2+^ (Fig. 5B) was immediately followed by arrest of mechanical activity; resumption of activity after wash out was accompanied by post-rest potentiation. This response pattern is a fingerprint of cardiac EC-coupling [23]. Nifedipine caused a gradual decrease in twitch amplitude, compatible with progressive Ca^2+^ channel blockade, before contraction subsided. Blockade of either RyRs (ryanodine), or IP3R (2-APB), caused sudden arrest, which points to involvement of SR release channels in the pacemaking mechanism underlying spontaneous activity.

### Embryoid Body Formation Without DMSO and Treatment with pre-miRNA

**Figure 6:**
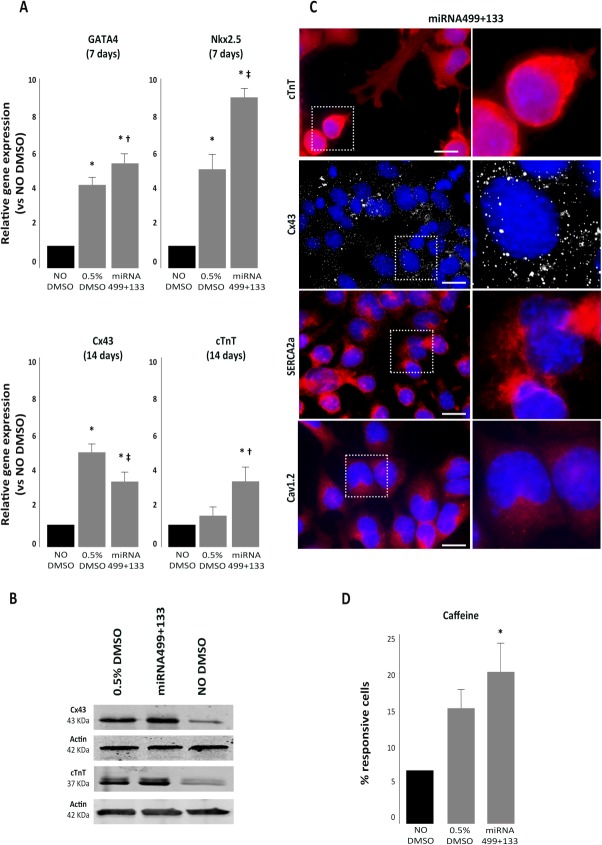
Embryoid bodies (EB) treated with pre-miRNA in the absence of DMSO. (A): Real-time PCR for “early” genes GATA4 and Nkx2.5 or “late” cardiac genes Cx43 and cTnT (*, *p* < .001 vs. NO DMSO; †, *p* < .01 vs. 0.5% DMSO; ‡, *p* < .001 vs. 0.5% DMSO). (B): Western blot analysis of Cx43 and cTnT. (C): ICC on EB treated with pre-miRNA133 in combination with pre-miRNA499: cTnT is shown in red, Cx43 in white, Serca2a in red, and Cav1.2 in red. Scale bar = 25 μm. (D): Percentage of cells responsive to caffeine. Comparison between untreated EB and EB exposed to premiRNA499 plus premiRNA133 or 0.5% DMSO (*, *p* < .001 vs. all conditions).

To strengthen our observation, we aimed to test whether treatment with miRNA499 plus miRNA133 in the absence of DMSO exposure was sufficient to trigger cardiac differentiation. Real-time PCR analysis showed that also in P19 cells not exposed to DMSO, treatment with miRNA499 and miRNA133 upregulated GATA4 (+4.9-fold, *p* < .001), Nkx2.5 (+8.7-fold, *p* < .001), Cx43 (+3.4-fold, *p* < .001), and cTnT (+2.9-fold, *p* < .001) (Fig. 6A). Importantly, the expression of GATA4, Nkx2.5, and cTnT was significantly higher after treatment with miRNAs compared with DMSO, considered so far the best inducer of cardiac differentiation when using P19 cells. Moreover, both the atrial marker Mlc.2A (Supporting Information Fig. S3C) and the ventricular marker IRX4 (Supporting Information Fig. S3D) were upregulated in P19 cells treated with pre-miRNA499 plus 133.

Of extreme relevance, WB analysis showed that the combination of miRNA499 plus miRNA133 upregulated the protein expression of both Cx43 and cTnT (Fig. 6B). ICC further confirmed that miRNA499 and miRNA133 coexpression was able to induce the expression of cardiac-specific proteins like cTnT, Cx43, Serca2a, and Cav1.2 (Fig. 6C) even in the absence of DMSO. Finally, functional analysis showed that the percentage of responsive EB grown without DMSO but transfected with pre-miRNA499 and pre-miRNA133 did not significantly differ from the percentage of EB grown in the presence of 0.5% DMSO (Fig. 6D).

### The Synergic Effect of miRNA499 and miRNA133 on AMSC

In order to confirm the synergic action of miRNA499 with miRNA133, we tested this combination also in AMSC. We performed real-time PCR to analyze the expression of early cardiac genes (GATA4 and Nkx2.5) on RNA extracted 7 days after treatment with miRNA precursors, and late cardiac genes (Cx43 and cTnT) at day 14.

**Figure 7:**
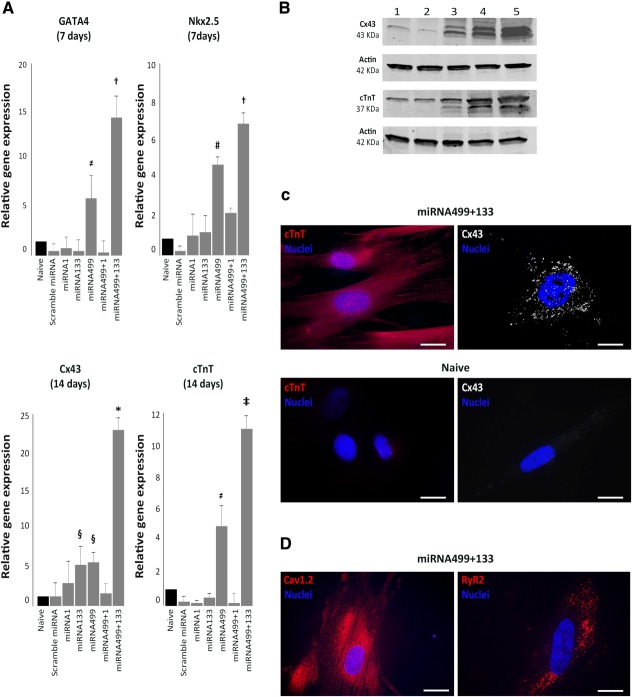
Amniotic mesenchymal stromal cells (AMSC) differentiation using miRNA499 and miRNA133 precursors. (A): Real-time PCR of “early” genes (GATA4 and Nkx2.5) and “late” cardiac genes (Cx43 and cTnT) (≠, *p* < .05 vs. naïve, scramble miRNA, miRNA1, miRNA133, and miRNA499 + 1; †, *p* < .001 vs. naïve, scramble miRNA, miRNA1, miRNA133, and miRNA1 + 499; #, *p* < .01 vs. naïve, scramble miRNA, miRNA133, miRNA1 + 499 and *p* < .05 vs. miRNA1; §, *p* < .01 vs. naïve, scramble miRNA and *p* < .05 vs. miRNA1 + 499; *, *p* < .001 vs. all conditions; ‡, *p* < .001 vs. naïve, scramble miRNA, miRNA1, miRNA133, miRNA1 + 499 and *p* < .01 vs. miRNA499). (B): Western blot analysis of the structural cardiac proteins Cx43 and cTnT and the internal control actin extracted from naïve AMSC (1), treated with miRNA scramble (2), or miRNA133 (3), or miRNA499 (4), or with both miRNA499 and miRNA133 (5). (C): ICC for cTnT and Cx43 in AMSC treated with miRNA499+133 and in naive AMSC. (D): Cav1.2, RyR2 in AMSC after induction with pre-miRNA499 plus 133.

As already observed in P19 cells, the combination of miRNA499 with miRNA133 triggered the over-expression of both the nuclear transcription factor GATA4 (+13-fold, *p* < .001) and Nkx2.5 (+5.3-fold, *p* < .001) when compared with naïve AMSC and all the conditions tested, except miRNA499 alone (Fig. 7A). Indeed, also miRNA499 alone induced a significant over-expression of GATA4 (+5.8-fold, *p* < .05) and Nkx2.5 (+3.8-fold, *p* < .01), but to a significantly lower extent compared with the combination (Fig. 7A). After 14 days, Cx43 was significantly over-expressed in cells treated with miRNA133 or miRNA499 and cTnT was significantly higher in the miRNA499 group compared with naïve cells (Fig. 7A). However, the coexpression of miRNA499 and miRNA133 resulted in a significantly higher expression of both cardiac markers compared with the other conditions tested (Fig. 7A).

WB (Fig. 7B) and ICC (Fig. 7C,D) analysis confirmed that AMSC treated with miRNA499 and miRNA133 differentiated in cells expressing Cx43 and cTnT (Fig. 7B, 7C) but also Cav1.2 and Ryr2 (Fig. 7D).

## Discussion

In recent years, stem cell therapy for cardiac regeneration has undergone intense investigation [24]. The ideal goal of this strategy is to substitute necrotic or disfunctioning cardiac tissue with new competent CMC derived from stem cells. These new generated CMC, in order to contribute to cardiac function, should be fully differentiated and electrically integrated with the surrounding native cardiac tissue. The first descriptions of cell plasticity raised genuine enthusiasm and hope that just by transplanting undifferentiated bone marrow-derived stem cells into damaged heart it would have been possible to regenerate cardiac tissue [25]. However, several studies have demonstrated that it is not that straightforward [26]. In particular, not every bone marrow-derived stem cell type possesses the capacity to differentiate into CMC [26,27]. On the contrary, several investigators have shown that mesenchymal stromal cells can differentiate into cardiac-like cells [28,29] even though the efficiency is low [9] and chemical compounds or coculture systems are required [30,31]. Thus, we would need new tools in order to achieve more efficient cardiac regeneration. Recently, it has been suggested that certain miRNA are powerful regulators of cardiac differentiation processes [32], and it has been shown that miRNA1, miRNA133, and miRNA499 are highly expressed in muscle cells [32]. It has been shown that miRNA1 and miRNA133 are important regulators of embryonic stem cell (ESC) differentiation into CMC. For example, loss- and gain-of-function studies documented that miRNA1 modulates cardiogenesis and muscle gene expression in Drosophila [20]. Moreover, miRNA1 is upregulated upon induction of cardiac differentiation in mouse and human ESC and in adult cardiac-derived progenitors [18,19,33]. Consistently, the over-expression of miRNA1 in EB derived from human ESC can increase the expression of myosin heavy chain [33]. Likewise, miRNA1 triggers the differentiation of cardiac progenitor cells [18]. Although miRNA1 and miRNA133 are cotranscribed, the function of miRNA133 is different from miRNA1. In particular, miRNA133 seems more crucial in controlling cell proliferation by repressing serum response factor and cyclin D2 [17,24]. Further studies demonstrated that miRNA499 is highly enriched in cardiac committed adult progenitor cells [18] and human ESC [33]. Over-expression of miRNA499 induces the expression of myosin heavy-chain (MHC) and cardiac-specific transcription factors in mouse and human ESC [33], while the inhibition of miRNA499 impairs the cardiac differentiation process [18]. It is currently unknown whether the concomitant over-expression of miRNA1, miRNA133, and miRNA499 or if the combination of two of these miRNA would result in a synergistic action, further increasing the efficiency of cardiac differentiation. To test this hypothesis, we first used P19 cells, a well-known and well-established cell line, to study cardiac differentiation. Our results clearly showed that miRNA499 is a powerful activator of cardiac differentiation, particularly in comparison with miRNA1 and miRNA133. However, when we coexpressed miRNA499 together with miRNA133 the results were significantly and strikingly superior compared with the over-expression of miRNA499 alone. In particular, miRNA499 plus miRNA133 almost doubled the number of beating EB compared with miRNA499 alone. Most importantly, by simultaneously over-expressing miRNA499 and miRNA133 the number of P19 cells expressing cTnI was 30-fold greater compared with the standard differentiation protocol. In addition, the expression of genes encoding for cardiac-specific transcription factors, such as GATA4 and Nkx2.5, and cardiac-specific proteins, such as Cx43 and cTnT, was enhanced in cells treated with miRNA499 plus miRNA133. Gene expression analysis documented that our protocol results in the production of both atrial and ventricular myocytes, as testified by the over-expression of Mlc.2A and IRX4, two well-known differentiation markers [34]. WB and ICC analysis confirmed that cardiac proteins are indeed expressed at higher levels when P19 cells are cotransfected with miRNA499 plus miRNA133.

Importantly, functional characterization of contracting EB reinforced the evidence that miRNA499 and miRNA133 synergistically induce cardiac differentiation. Recordings of EB electrical activity demonstrated their dependency on sarcolemmal ion channels. In particular, untreated EB showed responses compatible with Ca^2+^-dependent electrical activity, typical of immature CMC, while Na^+^-dependent excitability was recorded in EB over-expressing miRNA499 and miRNA133. While this observation might suggest improved electrical maturation, we cannot exclude that the MEA technique is unsuitable to achieve an adequate sample size to test events occurring at low frequency. Therefore, the effect of miRNA499 and miRNA133 synergism on cardiogenic differentiation was further tested based on the notion that mature excitation-contraction coupling relies on the presence of Ryrs-operated intracellular Ca^2+^ stores. Myocyte excitation-contraction coupling relies on the presence of intracellular Ca^2+^ stores, from which Ca^2+^ is released through RyRs channel proteins. RyRs can be opened by caffeine; thus, evidence of caffeine-induced Ca^2+^ transients implies the presence of a functional muscle-specific subcellular Ca^2+^ compartment. Coexpression of miRNA499 and miRNA133 sharply increased the proportion of caffeine-responsive cells. Clear-cut expression of cardiac EC-coupling proteins in the batch of EB in which caffeine responsiveness was detected provides molecular confirmation of the functional evidence. Whereas caffeine responsiveness does not differentiate cardiac from skeletal muscle, the absence of nicotine-induced Ca^2+^ responses is incompatible with skeletal muscle physiology [22]. Furthermore, the spontaneous mechanical activity response of miRNA499 and miRNA133 transfected cells to modulators of Ca^2+^ handling effectors (CaV, RyRs, and IP3R) is consistent with that expected for cardiac but not skeletal muscle. Such a response might still be compatible with smooth muscle, where IP3 and Ca^2+^-triggered EC-coupling coexist [35]. Nevertheless, isoforms of Ca^2+^ handling proteins specific to cardiac muscle were highly expressed.

To verify whether miRNA499 and miRNA133 exert their effects also on other cell types, we tested our protocol on AMSC. Gene and protein expression analysis showed that miRNA499 and miRNA133 are able to induce the differentiation of AMSC into cells expressing typical cardiac markers such as Nkx2.5, GATA4, cTnT, Cx43, Ryr2, and Cav1.2. It was impossible to document the same results using different combination of miRNAs, confirming that only the couple miRNA499/miRNA133 triggers the differentiation of MSC toward a cardiac-like phenotype. We were not able to document organization of cardiac proteins in sarcomeres. It is possible to speculate that an optimization of the protocol may lead to better results in terms of maturation of MSC-derived cardiac-like cells. A functional characterization of these cells, after optimization of the differentiation protocol, would also add important information on the role of miRNAs in cardiac cell maturation. However, this goes beyond the scope of this work, which was to demonstrate whether there is a synergistic effect in priming progenitor cells toward the cardiac lineage when two or more miRNAs are over-expressed.

## Conclusions

In summary, we demonstrated that miRNA499 and miRNA133 act in a synergic manner inducing P19 differentiation into CMC even in the absence of DMSO. CMC derived from P19 cells over-expressing miRNA499 and miRNA133 develop EC-coupling properties typical of mature CMC. Importantly for translational purposes, we have also shown that the same combination miRNA499 and miRNA133 is a powerful inducer of cardiac differentiation for human MSC. These results contribute to improve our understanding of cardiac differentiation from undifferentiated progenitor cells and may speed up the development of more effective cardiac regeneration therapies.
