# Supplementary figures and images for: Combination of miRNA499 and miRNA133 Exerts a Synergic Effect on Cardiac Differentiation
Source: Stem Cells. 2015 Mar 24;33(4):1187–99. doi: 10.1002/stem.1928 (PMC4409033; doi:10.1002/stem.1928)

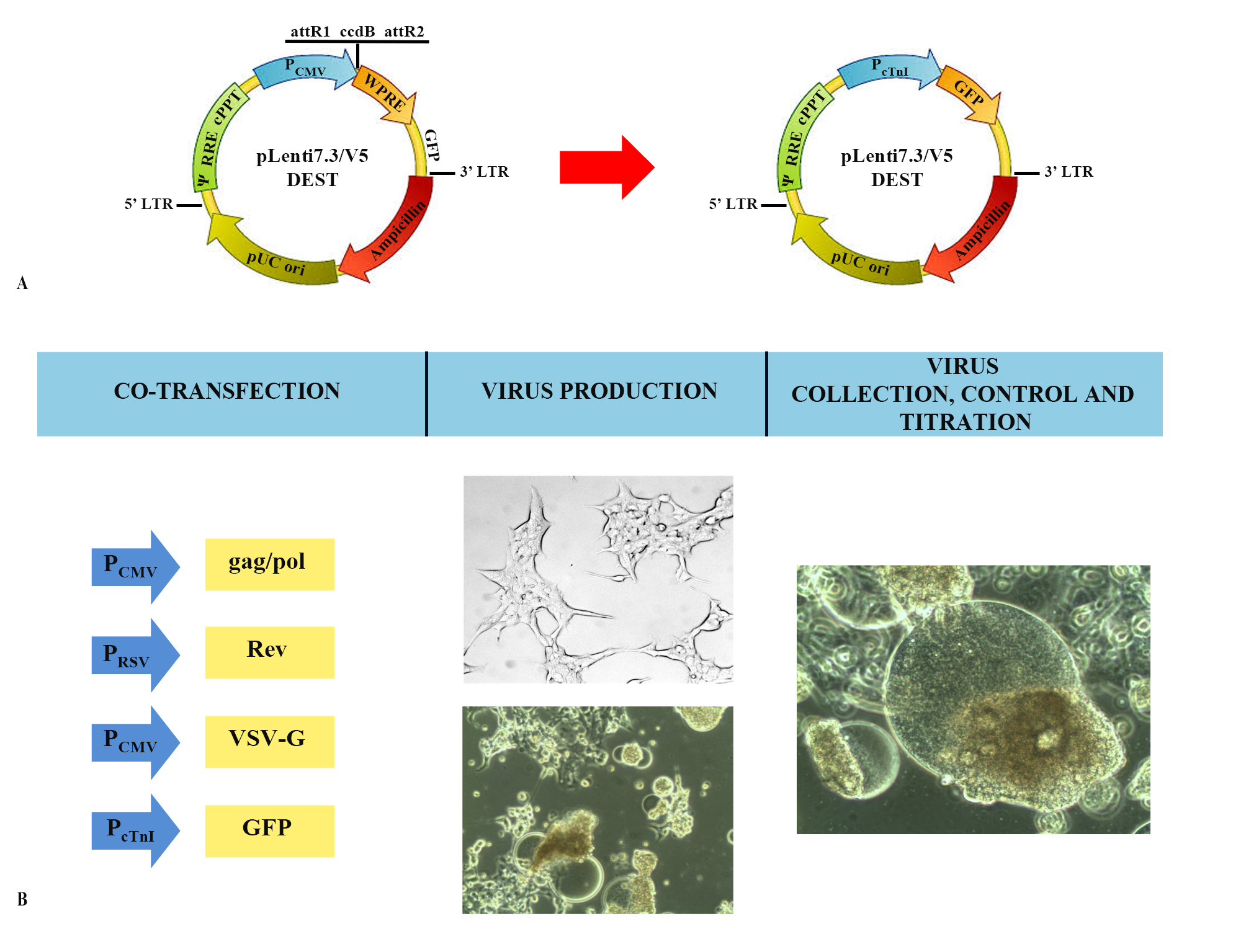

Supplement: Supplementary file 1 [file stem0033-1187-sd1.tif]

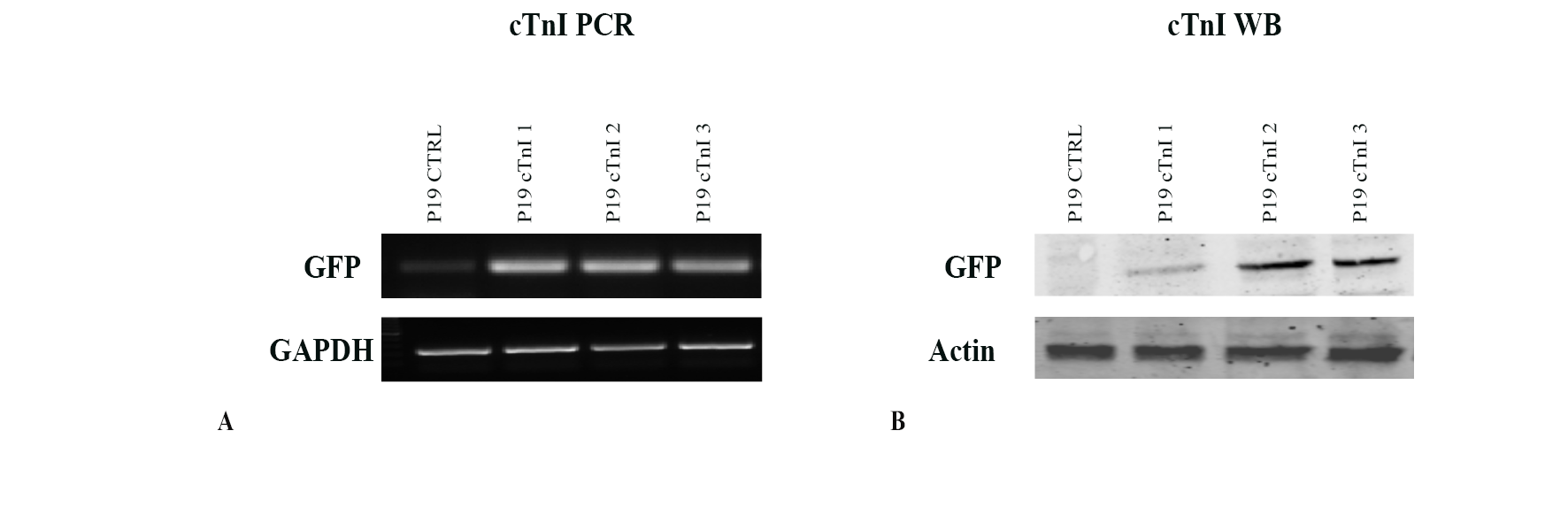

Supplement: Supplementary file 2 [file stem0033-1187-sd2.tif]

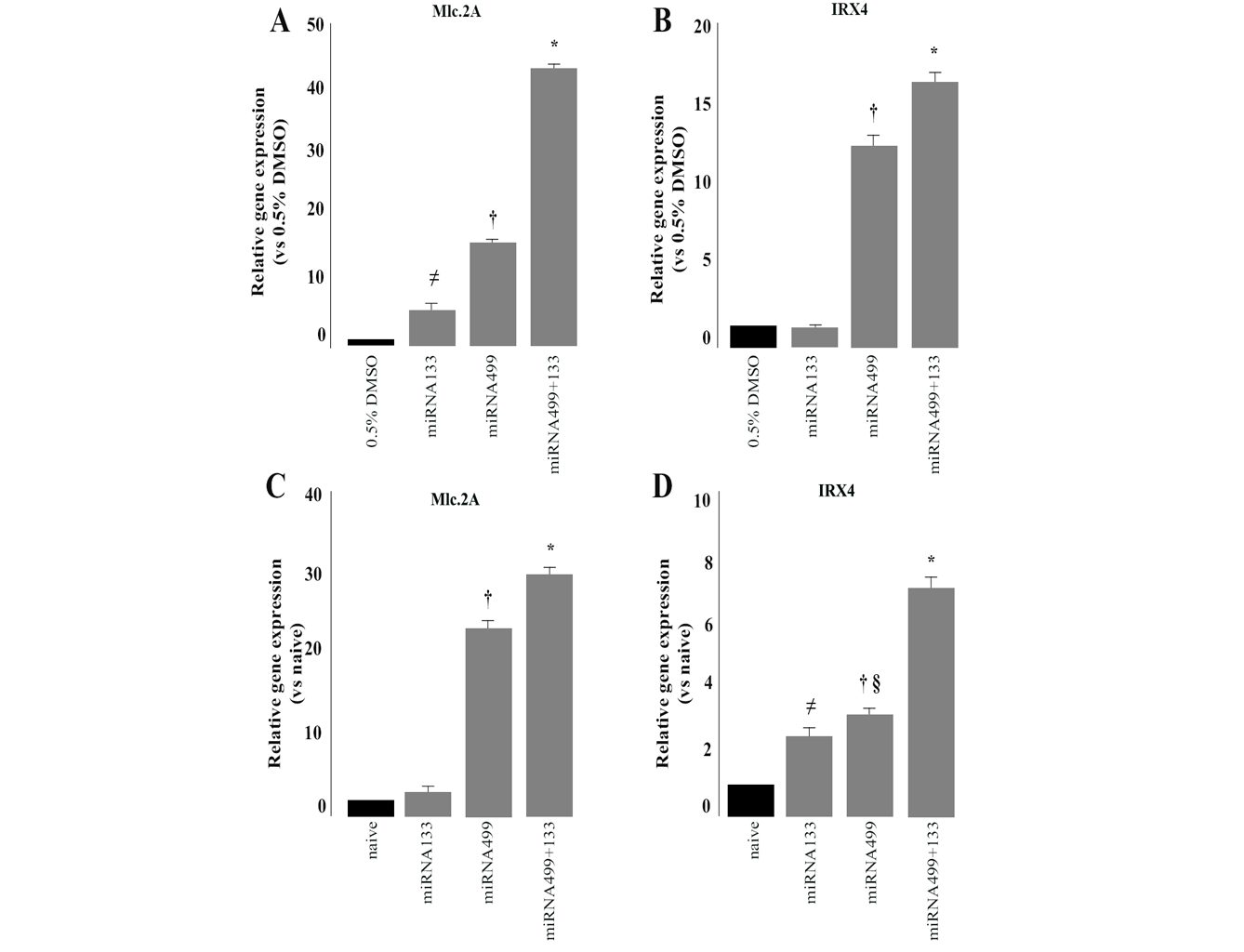

Supplement: Supplementary file 3 [file stem0033-1187-sd3.tif]

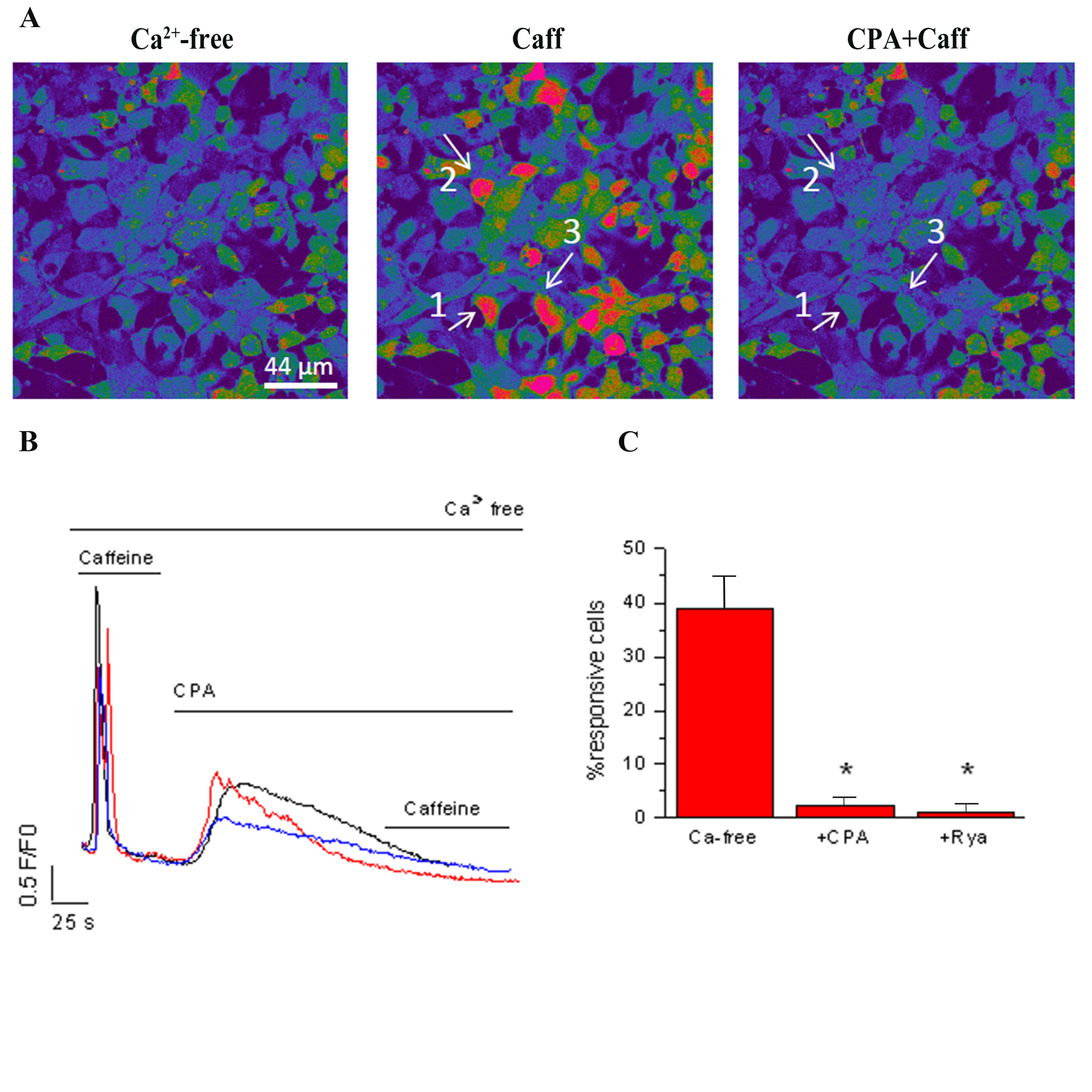

Supplement: Supplementary file 4 [file stem0033-1187-sd4.tif]

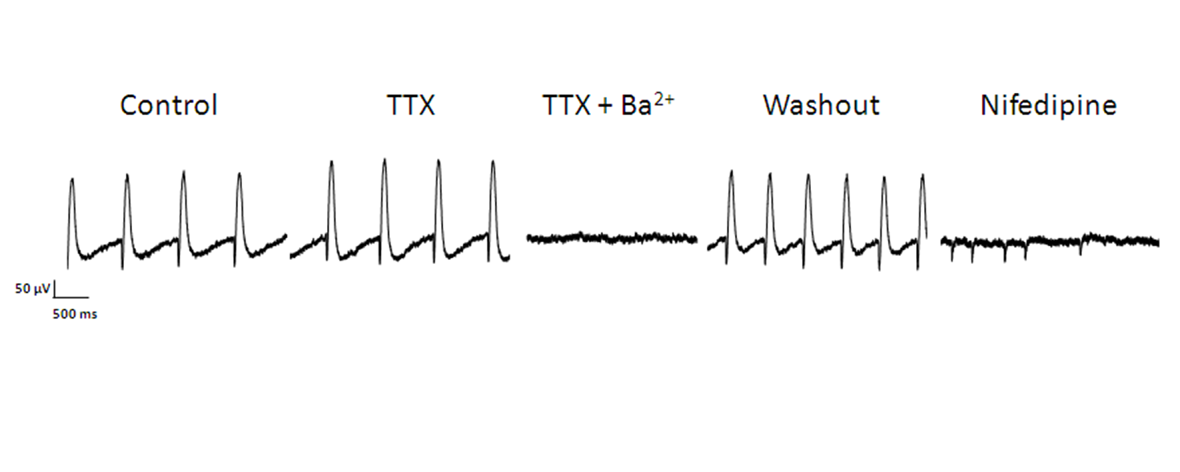

Supplement: Supplementary file 5 [file stem0033-1187-sd5.tif]
