# Supplementary material for: Combination of miRNA499 and miRNA133 Exerts a Synergic Effect on Cardiac Differentiation
Source: Stem Cells. 2015 Mar 24;33(4):1187–99. doi: 10.1002/stem.1928 (PMC4409033; doi:10.1002/stem.1928)
Supplement: Supplementary file 6 [file stem0033-1187-sd6.doc]

**SUPPLEMENTARY DATA**

**Preparation of cTnI lentiviral plasmid and virus.**

The cTnI sequence was cloned in a pZac backbone using the endonucleases XbaI and BamHI (Promega, [Fitchburg, Wisconsin](http://en.wikipedia.org/wiki/Fitchburg,_Wisconsin), US); the complete sequence of the promoter was isolated and purified. The expression vector pLenti7.3/V5-DEST plasmid (Invitrogen, Life Technologies, [Carlsbad](http://en.wikipedia.org/wiki/Carlsbad,_California), California, [US](http://en.wikipedia.org/wiki/United_States)A) was digested with ClaI (Promega, [Fitchburg, Wisconsin](http://en.wikipedia.org/wiki/Fitchburg,_Wisconsin), USA) and KpnI (Sigma Aldrich, [St. Louis, Missouri](http://en.wikipedia.org/wiki/St._Louis,_Missouri) [US](http://en.wikipedia.org/wiki/United_States)A) to obtain a linearized plasmid with all the necessary sequences for virus production, such as Long Terminal Repeats (LTR), Polypurine Tract (cPPT), Woodchuck Posttranscriptional Regulatory Element (WPRE) and φ, but without the constitutive promoter and a GFP under the control of the SV40 promoter (**Supplementary Figure 1A**). The cTnI promoter and the linearized vector were used for a ligation protocol with T4 DNA Ligase (Promega, [Fitchburg, Wisconsin](http://en.wikipedia.org/wiki/Fitchburg,_Wisconsin), USA) following the manufacturer’s instructions. After PCR, digestion and sequencing of the new final expression lentiviral vector, we used the constructs with three other plasmids (Virapower Mix, Invitrogen, Life Technologies, [Carlsbad](http://en.wikipedia.org/wiki/Carlsbad,_California), California, [US](http://en.wikipedia.org/wiki/United_States)A) to produce lentiviral particles in a packaging cell line called 293FT (Invitrogen, Life Technologies, [Carlsbad](http://en.wikipedia.org/wiki/Carlsbad,_California), California, [US](http://en.wikipedia.org/wiki/United_States)). At day 0, 293FT were plated in 175 cm^2^ flasks at a density of 2·10^5^ cells. At day two, the cells were washed twice with PBS and culture medium without antibiotics was added. At day three, cells were co-transfected over-night with Lipofectamine 2000 (Invitrogen, Life Technologies, [Carlsbad](http://en.wikipedia.org/wiki/Carlsbad,_California), California, [US](http://en.wikipedia.org/wiki/United_States)A), 5 μg pLenti7.3 with the cTnI promoter and GFP tag, 10 μg Virapower Mix (**Supplementary Figure 1B**). Forty-eight hours after co-transfection cellular debris was removed, virus particles were collected and concentrated with PEG-it Virus Precipitation Solution 5X (System Biosciences, [Mountain View, Silicon Valley, California](http://www.evi.com/q/facts_about__mountain_view_santa_clara_ca)). Lentivirus expressing the reporter gene GFP under the control of the cTnI promoter was quantified with the p24 ELISA kit (Clontech, Mountain View, Silicon Valley, California) and used to transfect P19 with a MOI of 3 TU/mL. Cardiogenic differentiation was evaluated by measuring the activity of the promoter of the cardiac specific cTnI. PCR was performed on transduced cells in order to confirm the integration of the cTnI promoter sequence into the P19 cell genome. Briefly, transduced P19 cells were treated with the differentiation protocol by adding 0.5% DMSO. EB were plated and maintained at 37 °C, 5% CO_2_ for 14 days. RNA and proteins were extracted and then used for PCR and WB analysis of GFP (**Supplementary Figure 2**).

**FACS analysis.**

Transduced cells were analyzed utilizing a specific FITC filter with the FACSCalibur flow cytometer 14 days after transduction and DMSO treatment. In detail, to analyze GFP expression, both GFP^+^ and GFP­^-^ EB were gently collected and placed in sterile tubes. The harvested cells were treated with tryspin for 5 minutes at 37 °C, to enhance the dissociation of EB into single cells. Tryspin was inactivated by adding 1:2 culture medium with 10% FBS. The cells were washed tree times with 1X PBS and finally analyzed.

**RNA extraction, reverse transcription, PCR and Real Time PCR protocols.**

Total RNA was isolated from EB using TRIZOL® reagent (Invitrogen, Life Technologies, [Carlsbad](http://en.wikipedia.org/wiki/Carlsbad,_California), California, USA) and following the manufacturer’s instructions. Briefly, cells were detached from culture dishes with a scraper and using TRIZOL® solution. The lysed cells were collected and chloroform was added. After 10 minutes incubation, samples were centrifuged for 15 minutes at 4°C, 12000 *g*. The upper aqueous phase was collected and mixed with 100% isopropanol and incubated at -30°C over-night. The mixture was centrifuged for 30 minutes at 4°C, 12000 *g*, the white, opalescent RNA pellets were washed with 75% ethanol and centrifuged at 4°C, 7500 *g* for 5 minutes. Finally, the supernatant was eliminated, and the pellet was air-dried, resuspended in DEPC water and quantified.

RNA was reverse transcribed for subsequent PCR with the following protocol: 1 μL OdT (Promega, [Fitchburg, Wisconsin](http://en.wikipedia.org/wiki/Fitchburg,_Wisconsin), US) and 2 μL dNTPs each 10 mM (Rovalab, Teltow Germany) were added to 500 ng RNA diluted with DEPC water to a total volume of 9 μL. The resulting mix was incubated for 5 minutes at 65°C. After incubation on ice for 5 minutes, a second mix composed of 4 μL 5X First Strand Buffer (Invitrogen, Life Technologies, [Carlsbad](http://en.wikipedia.org/wiki/Carlsbad,_California), California, USA), 2 μL 0.1M DTT (Invitrogen, Life Technologies, [Carlsbad](http://en.wikipedia.org/wiki/Carlsbad,_California), California, [USA](http://en.wikipedia.org/wiki/United_States)) and 1 μL RNase inhibitor, 20 units/μL (Applied Biosystems, Life Technologies, [Carlsbad](http://en.wikipedia.org/wiki/Carlsbad,_California), California, USA) was added to the samples and incubated for 2 minutes at 42°C. Finally, 1 μL SuperScriptII Reverse Transcriptase (Invitrogen, Life Technologies, [Carlsbad](http://en.wikipedia.org/wiki/Carlsbad,_California), California, [US](http://en.wikipedia.org/wiki/United_States)) was added and the samples were incubated for 50 minutes at 45°C, followed by 15 minutes at 72°C. To ensure the degradation of the remaining RNA, 1 μL RNase ONE Ribonuclease (Promega, [Fitchburg, Wisconsin](http://en.wikipedia.org/wiki/Fitchburg,_Wisconsin), USA) was added and incubated for 20 minutes at 37°C.

For Real Time amplification of the cardiac genes: GATA4, Nkx2.5, Tbx5, Cx43 and cTnT, 100 ng of RNA diluted with DEPC water to 4 μL were retro-transcribed preparing a single mix with: 2 μL 2.5 mM dNTPs (Rovalab, Teltow Germany), 1 μL 10X RT Buffer (Applied Biosystems, Life Technologies, [Carlsbad](http://en.wikipedia.org/wiki/Carlsbad,_California), California, [US](http://en.wikipedia.org/wiki/United_States)), 0.0625 μL MultiScribe Reverse Transcriptase 50 units/μL (Applied Biosystems, Life Technologies, [Carlsbad](http://en.wikipedia.org/wiki/Carlsbad,_California), California, [US](http://en.wikipedia.org/wiki/United_States)), 0.2 μL Rnase inhibitor 20 units/μL (Applied Biosystems, Life Technologies, [Carlsbad](http://en.wikipedia.org/wiki/Carlsbad,_California), California, [US](http://en.wikipedia.org/wiki/United_States)) and 0.5 μL random hexamers (Invitrogen, Life Technologies, [Carlsbad](http://en.wikipedia.org/wiki/Carlsbad,_California), California, [US](http://en.wikipedia.org/wiki/United_States)). The samples were incubated at 25°C for 10 minutes, 48°C for 30 minutes and 95°C for 5 minutes.

For Real Time amplification of miRNA sequences, total RNA was reverse transcribed using the Taqman miRNA reverse stem-loop primers (Applied Byosistems, Life Technologies, [Carlsbad](http://en.wikipedia.org/wiki/Carlsbad,_California), California, USA) with a Mastercycler EPgradients (Eppendorf, [Hamburg, Germany](http://en.wikipedia.org/wiki/Hamburg,_Germany)). Briefly, 5 ng RNA were diluted with DEPC water to 5 μL, to which 0.15 μL 10 mM dNTPs (Rovalab, Teltow Germany), 1.5 μL 10X RT buffer, (Applied Biosystems, Life Technologies, [Carlsbad](http://en.wikipedia.org/wiki/Carlsbad,_California), California, [US](http://en.wikipedia.org/wiki/United_States)), 1 μL MultiScribe Reverse Transcriptase 50 units/μL (Applied Biosystems, Life Technologies, [Carlsbad](http://en.wikipedia.org/wiki/Carlsbad,_California), California, [US](http://en.wikipedia.org/wiki/United_States)), 0.19 μL 20 units/μL Rnase inhibitor (Applied Biosystems, Life Technologies, [Carlsbad](http://en.wikipedia.org/wiki/Carlsbad,_California), California, [US](http://en.wikipedia.org/wiki/United_States)) and 3 μL 5X specific primers (Applied Biosystems, Life Technologies, [Carlsbad](http://en.wikipedia.org/wiki/Carlsbad,_California), California, [US](http://en.wikipedia.org/wiki/United_States)) depending on the miRNA, were added. The samples were incubated at 16°C for 30 minutes, 42°C for 30 minutes and 85°C for 5 minutes.

The PCR protocol used was the following: 5 μL 5X Green GoTaq Reaction Buffer (Promega, [Fitchburg, Wisconsin](http://en.wikipedia.org/wiki/Fitchburg,_Wisconsin), USA), 2 μL 10 μM forward primer and 2 μL 10 μM reverse primer, 0.5 μL 10 mM dNTPs (Rovalab, Teltow Germany), 13.4 μL DEPC water, 0.2 μL GoTaq DNA Polymerase 5 units/μL (Promega, [Fitchburg, Wisconsin](http://en.wikipedia.org/wiki/Fitchburg,_Wisconsin), USA) and 2 μL cDNA were mixed together. The samples were then incubated at 95°C for 2 minutes, followed by 33 cycles of denaturation at 95°C for 45 seconds, annealing at 56°C for 45 seconds and elongation at 72°C for 1 minute. Finally, the samples were elongated at 72°C for 10 minutes. The PCR products were analyzed with 2% GellyPhor (Celbio Euroclone Group, Milan, Italy) agarose gel and stained with ethidium bromide.

Real Time PCR of cardiac genes was performed by adding the following to 1 μL of cDNA: 12.5 μL Power SYBR Green PCR Master Mix (Applied Biosystems, Life Technologies, [Carlsbad](http://en.wikipedia.org/wiki/Carlsbad,_California), California, USA), 1.5 μL 300 mM forward primer and 1.5 μL 300 mM reverse primer, 8.5 μL DEPC water. The protocol consisted in a first incubation at 95°C for 10 minutes, 45 cycles of 95°C for 15 seconds and 60°C for 1 minute and finally a dissociation step at 95°C for 15 seconds, 60°C for 15 seconds and 95°C for 15 seconds.

Real Time PCR for specific miRNA sequences was performed by adding to 1.33 μL cDNA: 1 μL 20X specific primer (Applied Biosystems, Life Technologies, [Carlsbad](http://en.wikipedia.org/wiki/Carlsbad,_California), California, USA), 10 μL TaqMan Universal PCR Master Mix, 10 μL No AmpErase UNG (Applied Biosystems, Life Technologies, [Carlsbad](http://en.wikipedia.org/wiki/Carlsbad,_California), California, USA) and 7.67 μL DEPC water. Samples were incubated at 95°C for 10 minutes, 95°C for 15 seconds and 60°C for 1 minute, for 45 cycles.

**Protein extraction and Western Blot.**

Total proteins were extracted by lysing cells transfected with pre-miRNA for seven and 14 days, with ice-cold RIPA buffer (25 mM Tris pH 7.4, 150mM NaCl, 1mM EDTA, 1% Igepal CA 630.1% sodium deoxycholate, 0.01% sodium dodecyl sulfate (SDS) supplemented with protease inhibitor cocktail kit (Halt Protease Inhibitor Cocktail, Thermo Scientific, Rockford IL, USA) for 30 minutes at 4°C under agitation. Cell lysates were then centrifuged at 9500 *g* for 15 minutes at 4°C and protein concentrations were determined with the bicinchoninic acid assay (BCA) kit (Sigma-Aldrich, St. Louis, MO, USA) according to the manufacturer’s instructions. Proteins were boiled with 5X sample buffer (0.3M Tris–HCl, pH 6.8, 2% SDS, 30% glycerol, 20% (v/v) β-mercaptoethanol, 0.2% (w/v) bromophenol blue), separated on 12.5% SDS-PAGE gels (acrylamide/bis solution 37.5:1, Tris/HCl 1.5 M pH 8.8, 10% SDS, 1% Temed and 10% ammonium persulfate in H_2_O) and transferred onto a nitrocellulose membrane with a semi-dry transfer system (BioRad Laboratories, Hercules, CA, USA). After blocking in Odyssey® Blocking Buffer (LI-COR Biosciences, [Lincoln, NE, USA](http://en.wikipedia.org/wiki/Lincoln,_Nebraska)) diluted 1:2 with PBS for 1 hour at room temperature. Membranes were then rinsed and incubated over-night at 4ºC with the appropriate primary antibody diluted in Odyssey® Blocking Buffer diluted 1:2 with PBS and supplemented with 0.2% Tween 20, with gentle shaking. Anti-cardiac troponin T isoform (cTnT) mouse monoclonal antibody (1F11, dilution 1:500) and anti-connexin 43 (Cx43) mouse monoclonal antibody (CXN-6, dilution 1:500), both from AbCam (Cambridge, UK), were used as primary antibodies. Beta actin mouse monoclonal antibody (AbCam, Cambridge, UK, dilution 1:400) was used as a loading control. Membranes were then washed in Odyssey® Wash Buffer (PBS and 0.1% Tween 20) to remove unbound primary antibody and subsequently incubated for 1 hour with the specific IRDye secondary antibody (700CW or 800CW) at room temperature. After 3 additional washes in Odyssey® Wash Buffer and a final wash in PBS, bands were visualized with Odyssey® Infrared Imaging System (LI-COR Biosciences, [Lincoln, NE, USA](http://en.wikipedia.org/wiki/Lincoln,_Nebraska)).

**Immunocytochemistry protocols.**

Immuonocytochemistry (ICC) analysis was performed to assess the expression of cTnT and Cx43, sarcolemmal L-type voltage-dependent Ca^2+^ channels (alpha-1C subunit Cav1.2), [sarco](http://en.wikipedia.org/wiki/Sarcoplasmic_reticulum)plasmic reticulum (SR) Ca^2+^ [ATPase](http://en.wikipedia.org/wiki/ATPase) (cardiac isoform SERCA2a) and Ca^2+^ release channels (cardiac isoform RyR2). P19 cells were fixed with 4% paraformaldehyde (PFA) and permeabilized with 0.1% Triton X in PBS. Cells were washed twice and then blocked with bovine serum albumin (BSA) for 30 minutes at room temperature. Mouse monoclonal antibodies anti-cTnT (1F11, dilution 1:400), Cx43 (CXN-6, dilution 1:500), Cav1.2 (1:200) and RyR2 (1:200) and rabbit anti-SERCA2a monoclonal antibody (1:100), all from AbCam (Cambridge, UK), were used as primary antibodies. The cells were incubated 1 hour at 37°C with primary antibody diluted with BSA. Then, specific secondary antibodies (AlexaFluor 488 and 546 Molecular Probes from Invitrogen, Life Technologies, [Carlsbad](http://en.wikipedia.org/wiki/Carlsbad,_California), California, USA; dilution 1:500 in BSA) were used. Nuclei were counterstained with Hoechst33258 diluted 1:1000 in PBS for 10 minutes at room temperature. Microscopy analysis was performed using an inverted fluorescent microscope (Zeiss Axio Observer Z1), equipped with a system allowing obtainment of optical sections (ApoTome).

**Mesenchymal stem cell isolated from human amniotic membrane.**

Human term placentas were obtained from healthy donor mothers undergoing a caesarean section. The study was approved by the Ethical Committee of the Fondazione IRCCS Policlinico San Matteo (Pavia) and all the samples were collected after obtaining informed consent. Fragments of the amniotic membrane were digested using 0.25% trypsin-EDTA and subsequently in 50 U/ml collagenase (Sigma Aldrich, [St. Louis, Missouri](http://en.wikipedia.org/wiki/St._Louis,_Missouri) [US](http://en.wikipedia.org/wiki/United_States)) in Hank’s Balanced Salt Solution (HBSS) (Celbio EuroClone Group, Milan, Italy). Supernatant was collected and centrifuged at 200 g for 10 minutes. Harvested cells were cultured in polystyrene culture dishes (Corning Inc., Corning, NY, USA) at 37°C with an atmosphere of 95% air and 5% CO_2_ in α-Minimum Essential Medium (α-MEM; Celbio EuroClone Group, Milan, ITALY) supplemented with 10% Fetal Bovine Serum (FBS), 2 mmol/L L-glutamine, 100 U/ml penicillin and 100 mcg/ml streptomycin (all from Gibco, Life Technologies, Carlsbad, CA, US). Amniotic mesenchymal stem cells (AMSC) were selected by plastic-adherence and propagated in colture. AMSC were trypsinized after reaching 80% confluence, seeded at a standard density of 1000 cells/cm^2^ and propagated in culture in alpha-MEM supplemented with 10% FBS, 2 mmol/L L-glutamine, 100 U/ml penicillin and 100 U/mL streptomycin until passage 10. In order to confirm the mesenchymal origin of the obtained cells, the phenotype of AMSC was defined by FACS analysis and their capacity to differentiate into adipocytes and osteocytes was tested (data not shown).

**Cardiac differentiation of amniotic mesenchymal stem cells.**

AMSC were differentiated at passage three. Briefly, for each 100 mm Petri dish, 10^6^ cells were used for transfection protocol with 10 μL of miRNA499 (10 nM) and 18 μL of miRNA133 (5 nM) precursors diluted in 2.5 mL Opti-MEM I medium. The transfection protocol was the same used for P19 cells.
