# Supplementary material for: Combination of miRNA499 and miRNA133 Exerts a Synergic Effect on Cardiac Differentiation
Source: Stem Cells. 2015 Mar 24;33(4):1187–99. doi: 10.1002/stem.1928 (PMC4409033; doi:10.1002/stem.1928)
Supplement: Supplementary file 7 [file stem0033-1187-sd7.docx]

|  |  |
| --- | --- |
| GFP F | 5’-ATGGCCACAACCATGGTGAGCAAG-3’ |
| GFP R | 5’-CTTGTACAGCTCGTCCATGCCGAG-3’ |
| GATA4 F | 5’-TCCAGCCTGAACATCTACCC-3’ |
| GATA4 R | 5’-TGTGTGTGAAGGGGTGAAAA-3’ |
| Nkx2.5 F | 5’-GTAGGGAAAGAGCCCGTTTG-3’ |
| Nkx2.5 R | 5’-TCTGAGGGACAGGGCATAGT-3’ |
| Tbx5 F | 5’-CAAACTCACCAACAACCACCT-3’ |
| Tbx5 R | 5’-TTTGTGCATGGAGTTCAGGA-3’ |
| Cx43 F | 5’-CTTCCTGCTGATCCAGTGGT-3’ |
| Cx43 R | 5’-CAGGGATCTCTCTTGCAGGT-3’ |
| cTnT F | 5’-GCCAAAGATGCTGAAGAAGG-3’ |
| cTnT R | 5’-ACCAAGTTGGGCATGAAGAG-3’ |
| GAPDH F | 5’-ATCACTGCCACCCAGAAGAC-3’ |
| GAPDH R | 5’-GGATGCAGGGATGATGTTCT-3’ |
| Mlc.2A F | 5’-CACTCCCTAAACTCGGCCAT-3’ |
| Mlc.2A R | 5’-CAGTACCCCTAGTGTCCCCA-3’ |
| IRX4 F | 5’-AAGTAGAGCTCACCGCCAAC-3’ |
| IRX4 R | 5’-CCTCCCTCTGAAACAGGCAA-3’ |
| Pre-miRNA1 | 5’-UGGAAUGUAAAGAAGUAUGUAU-3’ |
| Pre-miRNA133 | 5’-UUUGGUCCCCUUCAACCAGCUG-3’ |
| Pre-miRNA499 | 5’-UUAAGACUUGCAGUGAUGUUU-3’ |
